# Mammalian sphingosine kinase (SphK) isoenzymes and isoform expression: challenges for SphK as an oncotarget

**DOI:** 10.18632/oncotarget.16370

**Published:** 2017-03-18

**Authors:** Diana Hatoum, Nahal Haddadi, Yiguang Lin, Najah T. Nassif, Eileen M. McGowan

**Affiliations:** ^1^ School of Life Sciences, University of Technology Sydney, Ultimo, Sydney, NSW 2007, Australia

**Keywords:** sphingosine kinase, isoenzymes, isoforms, cancer, sphingosine-1-phosphate

## Abstract

The various sphingosine kinase (SphK) isoenzymes (isozymes) and isoforms, key players in normal cellular physiology, are strongly implicated in cancer and other diseases. Mutations in SphKs, that may justify abnormal physiological function, have not been recorded. Nonetheless, there is a large and growing body of evidence demonstrating the contribution of gain or loss of function and the imbalance in the SphK/S1P rheostat to a plethora of pathological conditions including cancer, diabetes and inflammatory diseases. SphK is expressed as two isozymes SphK1 and SphK2, transcribed from genes located on different chromosomes and both isozymes catalyze the phosphorylation of sphingosine to S1P. Expression of each SphK isozyme produces alternately spliced isoforms. In recent years the importance of the contribution of SpK1 expression to treatment resistance in cancer has been highlighted and, additionally, differences in treatment outcome appear to also be dependent upon SphK isoform expression. This review focuses on an exciting emerging area of research involving SphKs functions, expression and subcellular localization, highlighting the complexity of targeting SphK in cancer and also comorbid diseases. This review also covers the SphK isoenzymes and isoforms from a historical perspective, from their first discovery in murine species and then in humans, their role(s) in normal cellular function and in disease processes, to advancement of SphK as an oncotarget.

## INTRODUCTION

**Figure 1:**
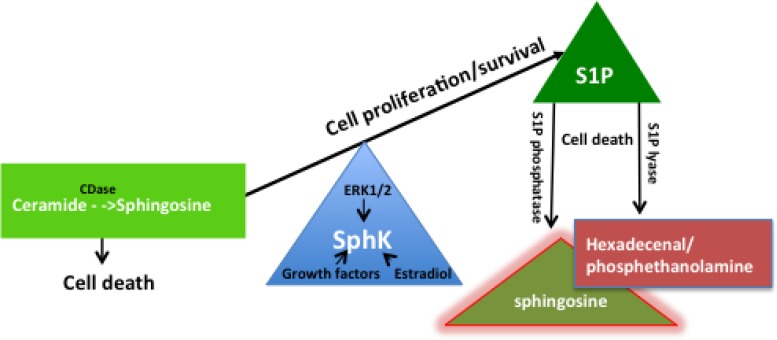
The sphingosine kinase (SphK) rheostat in the maintenance of cell proliferation and function SphK is a phospholipid enzyme that converts sphingosine to S1P. Through the repression of sphingosine by phosphorylation, SphK modulates the balance between S1P and sphingosine and ceramides to promote cell survival, normal cell proliferation and cell function. SphK has intrinsic catalytic activity to facilitate a housekeeping role in maintaining physiological levels of sphingosine and ceramide and is also stimulated by mitogens such as growth factors, estradiol, and ERK. On the other hand, S1P lyase irreversibly degrades S1P into hexadecenal and phosphoethanolamine while S1P phosphatase dephosphorylates S1P to sphingosine to maintain physiological levels of S1P and maintain homeostasis.

Sphingosine kinase (SphK), categorized as a bioactive lipid enzyme, is a central player in the sphingolipid rheostat [1–5]. The sphingolipid rheostat was first coined in the mid-nineties to describe the repression of ceramide-mediated programmed cell death through the conversion of sphingosine, a metabolite of ceramide, to sphingosine-1-phosphate (S1P) [5–7]. In this role, SphK modulates the balance between S1P, sphingosine and ceramides to maintain physiological levels of sphingosine and ceramide [8–10]. Activity of SphK/S1P is enhanced through cytokines, hormones, and growth factor stimulation [10–14]. Thus SphK is the rate-limiting enzyme that maintains the level of S1P for cell survival and normal cell proliferation and function. Conversely, S1P is enzymatically degraded by S1P lyase to maintain the level of S1P at normal physiological levels (Figure 1) [8, 15]. As will be described in this review, SphK has two major isoenzymes (isozymes), SphK1 and SphK2, and each isozyme is expressed as a number of isoforms [16–18]. Differences in conformation and dimerization properties, in addition to the varying subcellular localizations of SphK isozymes and isoforms, contribute to the diversity in SphK functions. SphK isozymes have some redundancy and compensatory functions in “normal” physiology, as described in mouse models. SphK knockout mice with deletion of either isozyme show no obvious phenotypic abnormalities [19–21]. Deletion of both isozymes results in embryonic fatality [19].

Recently our attention has been drawn to the diversity in biological functions of the SphK1 and SphK2 isoforms whereby each isozyme has multiple isoforms differing only at the N-terminus [16, 22–24]. There is a strong suggestion that imbalances of SphK1 isoform abundance may play a crucial role in the pathophysiology of diverse diseases, may contribute to resistance to current anti-cancer drug therapies [25–27], and may have consequences for therapies targeting SphK1 and S1P in the presence of comorbid conditions [15, 28]. Importantly, emerging evidence suggests that although high expression of both SphK-1a and -1b isoforms are associated with oncogenicity, the aberrant expression of the isoforms may be important to the efficacy of anti-SphK1 drug therapies [25, 26, 28]. Thus SphK isoform function and role in normal physiology and disease initiation and progression certainly merit further examination. This review updates our current knowledge of the SphK isozymes and highlights recent findings on the two prominent isoforms, SphK1a and SphK1b, and potential implications for efficacy of SphK1 directed cancer drug administration and outcome.

## CELLULAR PHYSIOLOGICAL ROLES OF SPHK, S1P AND S1P RECEPTORS (S1PR)

The evolutionary conservation of SphK amino acid sequences from protozoa, zebrafish and mammals (illustrated in Supplementary Figure 1) and its ubiquitous presence in living tissues highlights the importance of the cellular signaling pathway mediated by SphK [10]. It is therefore undeniable that SphK/S1P signaling is indispensable for healthy cell maintenance [29]. One of the main functions across all species is the role of S1P as an intracellular second messenger regulating calcium mobilization, balancing cell growth and survival [10].

In mammals, SphK/S1P is pivotal for normal cellular physiology through roles in cell proliferation, survival, differentiation, motility and intracellular calcium regulation [30, 31]. SphK activation of S1P is critical for embryonic development including, directed cell movement, organogenesis, cardiac development, maturation of vasculature, cellular immunity, and protection against apoptosis [10, 32]. S1P signaling is also essential for neurogenesis, lymphocyte trafficking and vascular development [33, 34]. Ongoing diverse roles for SphK/S1P in cellular maintenance also include protecting the heart and brain from ischemic damage [35, 36], protecting mitochondrial function [35, 37], regulating insulin synthesis, muscle insulin resistance, reduced glucose levels, maintenance of blood vessels and decreasing inflammation [15].

SphK promotes S1P activation through the dual intra- and extra- cellular actions of S1P, referred to as the “inside-out” signaling of S1P [38, 39]. Extracellular S1P acts as a primary messenger, or ligand receptor, whereby its signaling is modulated through binding to one of five-cell surface S1P receptors (S1PR_1-5_), also referred to as S1P-specific G protein-coupled transmembrane receptors (GPCRs), and endothelial differentiation gene (EDG) receptors [30, 31, 40, 41]. Regulation of S1P signaling through these five G-protein coupled-receptors is important for normal development and vascular maturation [31, 41]. This family of S1PRs are differentially regulated in different cell types to exert different cellular responses to S1P activation. The S1P_1-3_ receptors are widely distributed whereas S1PR_4_ is expressed in lymphoid tissues and lung, and S1PR_5_ expression is restricted to the nervous tissue, each receptor having distinct, and overlapping intracellular signaling pathways [41]. Not all the S1PRs are expressed on cells at the same time, and may be differentially expressed depending on stage of maturation and activation of the cell [31]. In mouse models, S1PR_1_ is essential for embryonic vascular development [34], whereas S1PR_2_ and S1PR_3_ show no obvious phenotypic differences [42]. In addition to extracellular S1P acting through S1PRs, S1P can function independently of S1PRs inside the cell as a second messenger. As reviewed by Strub and colleagues [43] the type of SphK isozyme and the location within the cell are responsible for the ‘inside-out’ signaling of S1P in response to extracellular stimuli.

In summary, the activation and response to S1P varies in a cell- or tissue- specific manner resulting from the SphK isozyme expression, sub-compartment localization of these enzymes, and the differential expression of the S1P receptors. The importance of these specific interactions in cancer transformation will be discussed.

## SPHINGOSINE KINASE ISOENZYMES (ISOZYMES)

Previous studies have identified two SphK isozymes, SphK1 (SK1, SPK1) and SphK2 (SK2, SPK2), each with the ability to catalyze the phosphorylation of sphingosine to its pro-active form S1P [16, 22–24]. Both SphKs isozymes act through the same substrate, sphingosine, and exert intra- and extracellular actions through their ability to phosphorylate sphingosine to S1P [10]. The two isozymes have different tissue distribution with SphK1 being highly expressed in spleen, lung and leukocytes and SphK2 being highly expressed in liver and kidney [16]. SphK2 also appears much later in embryologic development compared to SphK1 [16]. As shown in murine models, knockout of both SphK isozymes is lethal, leading to embryonic fatality and SphK deficient mice present with defects in neural and vascular development [19].

The two isozymes are expressed from different chromosome locations: the gene encoding the SphK1 isozyme is located on chromosome 17 (17q25.2) and the gene encoding the SphK2 isozyme maps to chromosome 19 (19q13.2) [16]. The SphK2a and SphK1a isozymes, differ considerably in size (618 amino acids in length compared to 384 amino acids respectively) but, nonetheless, retain 45% overall identity and 80% similarity with comparable enzymic activity [16]. Although the two SphK isozyme protein sequences differ, they share five conserved domains involved in ATP binding and catalytic conversion of sphingosine to S1P [44, 45]. The size difference between the two isozymes result from additional amino acids in the N-terminal region, and a unique central proline-rich region which appears to coincide with the sphingosine binding region [46]. Studies of this SphK2 unique proline-rich region suggest it may confer some promiscuity to this isozyme in context of substrates it can phosphorylate [46]. This unique N-terminal region also allows for the targeting of isozyme selective inhibitors. Differences in the sphingosine binding region between the two SphK isozymes also allow SphK1 and SphK2 selective ATP competitive inhibitors to be developed therefore permitting isozyme-specific inhibitory therapeutic targeting [47]. SphK2 also contains a nine amino acid domain similar to the pro-apoptotic BH3 domain (Bcl2 homology domain 3), which is believed to be involved in the control of calcium levels in the cell, and in some culture conditions such as serum starvation [22].

**Figure 2:**
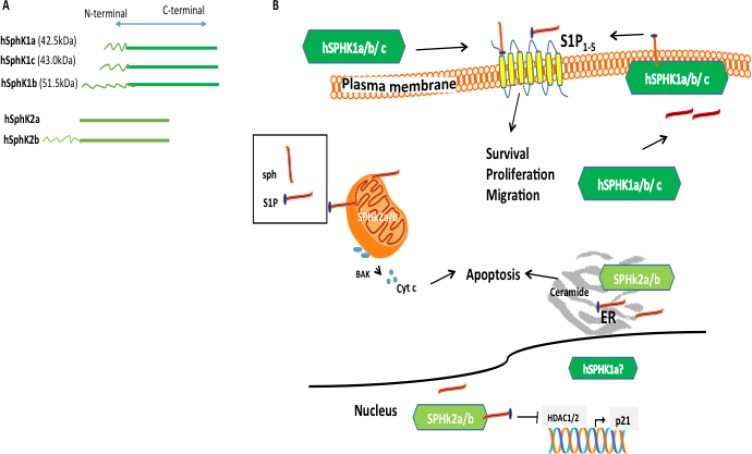
Subcellular distribution of SphK isozymes and function **A**. SphK is expressed as two isozymes designated SphK1 and SphK2. Each SphK isozyme has variant isoforms differing only at the N-terminus. **B**. The distinct functions of hSphK1 and hSphK2 isozymes are believed to be associated with subcellular localization. SphK2 isoforms are predominantly localized in the nucleus, mitochondria, and endoplasmic reticulum (ER) and produce S1P. While located at the ER, SphK phosphorylates sphingosine to S1P, conversely, S1P phosphatase removes the phosphate and S1P lyase degrades S1P to induce apoptosis. In the mitochondria, Sphk2 catalysis to produce S1P triggers the apoptotic pathway by activating BAK and Cyt C. Conversely, hSphK1 is predominantly located in the cytoplasm and translocates to the membrane upon activation. Upon activation, hSphK1a/b/c phosphorylates sphingosine to produce S1P and has an “inside/outside” mechanism whereby S1P is translocated outside the cell and can bind to S1P_1-5_ receptors (also referred to as G-coupled receptors) contributing mainly to cell survival. HSphK1 isoforms have also been found extracellular. In mice, SphK1 isoform b has been shown to be located to the plasma membrane and more susceptible to degradation, however, to-date, no distinction between hSphK1 isoform localization has been demonstrated in the different human cells and human tissue with the exception that hSphK1a has been shown to be distributed in the nucleus and cytoplasm in cell culture. Figure 2B has been adapted from [27].

Initial studies in SphK1 and SphK2 knockout mouse models describe opposing functions of overexpression of mSphK2 and mSphK1 isozymes, SphK1 is linked with pro-survival and cell maintenance functions, whereas SphK2, in contrast, has been linked with cell growth inhibition and enhanced cellular apoptosis [22]. Interestingly, this simplistic viewpoint has shifted [46] and recent emerging functions of SphK1 and SphK2, relevant to the newly emerging SphK-targeting cancer therapies, will be discussed. The different biological functions of the two SphK isozymes have been attributed to differing temporal and spatial regulation through post-translational modification and specific protein and lipid interactions [46], which allows for localized S1P production and provides downstream targets within the cell [22, 48]. This distinct intracellular sub-compartmentalization has been cited as the driving force underpinning their diverse biochemical roles ([48] and references therein). SphK1 is located mainly in the cytoplasm and the cell membrane whereas SphK2 is mainly located in the mitochondria, nucleus, and the endoplasmic reticulum (ER) (Figure 2) [37, 48–50]. Overexpression of SphK2 increases cytosolic free calcium, inducing apoptosis, which is partly attributed to the unique SphK2-specific pro-apoptotic BH3 domain, not present in SphK1 [22]. Established functional roles for SphK1 in actin remodeling, endocytic recycling and endocytic membrane trafficking to maintain plasma membrane homeostasis have been described [51]. Furthermore, SphK1 has an important role in neurotransmission and is highly enriched in nerve terminals, specialized subcellular compartments of exo-endocytosis [51].

Interestingly, despite the distinct properties and locations of SphK1 and SphK2 within the cell and mice with deletion of both isozymes is embryologically lethal, mice with deletion of either isozyme show no obvious abnormalities at birth, remain viable, have no obvious differences in longevity, and histological analysis reveals no major abnormalities of organ structure, [19, 52], and SphK1 knockout mice exhibit normal inflammatory response [20, 53], suggesting that there is some redundancy in SphK1 and SphK2 functions in “normal” physiology, and also indicates compensatory functions for both isozymes in the cell. This “compensatory” mechanism, whereby SphK1 and SphK2 complement each other's actions may be explained, in part, by the potential fluidity of the two isozymes allowing the SphKs isozymes to move between subcellular compartments [54, 55]. The proposed concept suggests that this partitioning of the isozymes into lipid microenvironments would allow SphK-isozyme specific catalytic conversion of sphingosine to S1P and effective binding to localized S1P receptors otherwise not normally accessible if under strict subcellular compartmentalization. Despite the importance of SphK in normal cellular function and the strong association between aberrant SphK/S1P signaling and diseases, the mechanisms whereby lipid kinases locate and act on their substrates, in general, is not well understood.

## INVOLVEMENT OF SPHK AND S1P IN THE PATHOGENESIS OF CANCER AND OTHER DISEASES

As key players in normal physiology and in disease, it is unsurprising that there is a large body of evidence demonstrating the contribution of an imbalance in the SphK/S1P rheostat to a plethora of pathological conditions including cancer, diabetes, inflammatory diseases, neurodegenerative diseases (Parkinson's and Alzheimer's), cardiovascular and liver diseases, which have been extensively reviewed [2, 3, 11, 15, 18, 48, 56–72]. Although removal of one of the major SphK isozymes is not fatal, as determined in mice with knockout of either SphK1 or SphK2, what these studies have revealed is that loss of one isozyme increases the risk of major health problems including altered vasculature formation [73, 74], impairment in wound healing [75], abnormal islet formation, which could lead to type 1 diabetes [76], and cardiac dysfunction (ischemic) [77], among other complications. Alternatively, overexpression of SphK1 and aberrations in the “inside-out” dual mechanism of SphK1/S1P activation have been reported as contributors to cancer and inflammation [38, 39, 56, 78, 79], and this is supported by studies in SphK1-deficient mice, for example, reduction of SphK1 reduced colon cancer development [80, 81].

Although initial studies focused on SphK2 as a pro-apoptotic protein more recent evidence describes dual functionality for SphK2: a protective function in cell maintenance as well as its characterized pro-apoptotic functions. As reported by Gaire [82], SphK2 is protective against neuronal ischemic injury, potentially protecting the junctional protein in the blood-brain barrier [36, 83–86]. Furthermore, S1P production by SphK2 potentially protects dopaminergic neurons, the major source of dopamine, and potentially provides some protection against Parkinson's disease through maintaining and promoting mitochondrial function [70]. Massberg and colleagues provide evidence demonstrating SphK2 expression, regulates platelet aggregation and that deletion of SphK2 leads to reduced arterial thrombosis after vascular injury, suggesting SphK2 is a potentially important target for protection against arterial thrombosis [73, 74]. Knockout SphK2 mouse models also revealed the importance of SphK2 in cardio protection and survival of the heart [87, 88].

This review is not intended to cover, in any depth, the ever-growing list of disorders and/or the substantial evidence presented for the involvement of SphK/S1P in health and disease. For this information we refer the readers to the cited references and the seminal papers listed in Table 1. However, the changes that occur in the SphK/S1P signaling pathways in normal compared to abnormal cells is still being unraveled and the involvement of SphK isozymes, isoforms, S1P and S1PR in the pathophysiology of cancer will be covered in more depth.

**Table 1:** Involvement of hSphK in the pathogenesis of cancer and other diseases

Disease	Disease Sub-Type	Reference(s)*
Cancer	Breast Prostate	[11, 14, 28, 89–97] [26, 98–106]
	Leukaemia	[107–111]
	Lung	[112–116]
	Pancreas	[117–120]
	Renal	[121]
	Colon	[80, 81, 122]
	Gastric	[123]
	Liver and bile ducts	[124, 125]
	Ovarian and Cervical	[126–130]
	Brain (Glioma)	[131–135]
	Melanoma	[136–139]
Neurodegenerative diseases	Parkinson's disease	[140–143]
	Alzheimer's disease	[142, 144–154]
	Amyotrophic lateral sclerosis (ALS)	[155]
	Dementia	[156]
Diabetes		[76, 157–175]
Inflammatory diseases	Asthma, Atherosclerosis, Inflammatory bowel disease, Edema Autoimmune disorders such as rheumatoid arthritis and multiple sclerosis	[176–196]
Osteoporosis		[197–201]
Cardiovascular		[163, 202–205]
Liver		[161, 205–213]
Renal		[214–218]

*This is not an exhaustive list of SphK references but includes the most relevant studies for each disease type.

## INTRACELLULAR AND EXTRACELLULAR TARGETS OF S1P REGULATED BY SPHK1 AND SPHK2 AND RELEVANCE TO CANCER

### Validation of SphK1 and SphK2 as targets for anti-cancer treatment

Disruption of the sphingolipid rheostat has been well reported as a determinant of initiation and progression of many cancers (Table 1) and a focus for anti-cancer treatments. Removal of SphK tips the balance from production of the pro-active lipid S1P, which is involved in cell proliferation, to the production of the inactive or less active ceramide lipid with pro-apoptotic functions (Figure 1). Extensive studies from mouse models and cancer cell lines have placed SphK1 as a target for cancer therapy. Studies using genetic knockdown of SphK1 and SphK2 in mouse colon cancer models revealed SphK1^−/−^ mice were significantly protected against colon cancer whereas this protection was not evident in SphK2^−/−^ mouse models [80]. Knockdown of SphK1 using siRNA in glioma and breast cancer cell models decreases S1P levels with an accompanying accumulation of ceramide resulting in increased apoptosis [134, 219]. Based on these initial findings SphK1 was identified as a prospective oncogenic target. Interestingly, hSphK2 ablation using RNA interference technology was shown to be more efficacious in inducing cell death [134, 220] validating both SphK1 and SphK2 as targets for anti-cancer treatment. Here we present an overview of hSphK1 and hSphK2 isozymes and an alternative perception of hSphK2 as a more efficient target for anti-cancer therapy compared to SphK1.

### Oncogenic properties or “non-oncogenic addiction” of SphK1/S1P

Overexpression of SphK1 has been reported as a driver for many types of cancer and contributes to chemotherapy resistance and poor patient outcome (Table 1 and references therein). Xia and colleagues first identified the oncogenic potential of SphK1 in 2000 [221], and evidence supporting a key role in neoplastic survival and growth has come from SphK1^−/−^ mouse models showing reduced tumor size [222] and increased expression of hSphK1 has been reported in many different cancer types (Table 1) [97, 223]. Although SphK1 is now categorized as an oncogene, there is no evidence associating carcinogenesis with the presence of genetic mutations of SphK1 [224]. It has been widely suggested that the oncogenic properties of SphK1 are associated with “gain of function” through the overexpression and hyper-activation of S1P mediated intra- and extracellular signaling, or “non-oncogenic” addiction, may be responsible for SphK-associated pathophysiology [225]. Non-oncogenic addiction refers to the over reliance of cancer cells on the SphK1 signaling pathway for survival [226]. Normal cells do not usually tolerate high levels of SphK1 and in some cases high SphK1 in normal cells has been reported to have an anti-proliferative effect [225]. Enhanced intra-cellular signaling through SphK1/S1P, independent of G-coupled receptors, was proposed as a mechanism responsible for hyper-proliferation and cell survival [227, 228]. However, increases in SphK1 and increases in SphK1 signaling mediated both intra and extra-cellular through a plethora of different molecules have been identified as participating in the “gain of function” leading to tumorigenesis and have been extensively reviewed in [18]. For example “gain of function” may occur through S1P stimulation of SphK1 mediated by agonists such as, growth factors, estradiol, insulin, cytokines and/or extracellular signaling kinases (ERK) [90, 91, 225, 229]. Estrogen stimulates SphK1 to promote breast cancer development [91, 93] and is also associated with breast cancer treatment resistance [230]. SphK1 mediates insulin-like growth factor binding protein-3 (IGFBP-3) growth factor signaling in breast cells, where overexpression of IGFBP-3 is associated with cancer progression [14]. Proinflammatory cytokines, immunoglobulin receptors, small GTPases, calcium and protein kinase activators, are all described as mediators of SphK1/S1P activity [18, 135]. For example, interleukin 1 (IL-1) stimulates SphK1 (not SphK2) in the brain contributing to chronic neuroinflammation and stimulation of malignant glial cells correlating with poor prognosis for patients with glioblastoma multiforme [135].

Although the overexpression of SphK1/S1P appears to be associated with promoting malignancy, it is becoming ever more evident that non-oncogenic addiction is also associated with aberrant localization and translocation of SphK1 post stimulation [231, 232]. SphK1 is located mainly in the cytosol in unstimulated cells and upon stimulation by agonists SphK1 is upregulated and translocates to the plasma membrane. For example, SphK1 localized in the cytoplasm has been demonstrated to interact with the tumor necrosis factor-(TNF) receptor associated factor 2 (TRAF2), enhancing S1P activity associated with pro-survival cell signaling of S1P [233, 234], although it is noted that the TNF-mediated response appears to be cell-type and cell context specific [235, 236]. It was in 2005 that Pitson and colleagues proposed that intra-cellular dysregulation of SphK1 phosphorylation and localization, rather than total increase in SphK1 levels, were key elements in the transformation of the malignant phenotype: i.e. subcellular-compartmentalization and translocation of SphK1 dictates cancer transformation [237]. All hSphK1 isoforms harbor a distinct phosphorylation site at Ser225, and phosphorylation of the SphK1-Ser225 site is important for translocation of SphK1 to the plasma membrane [55, 237]. Increased Ser225 phosphorylation of SphK1, as a direct consequence of extracellular ERK1/2-mediated phosphorylation at Ser225, was shown to enhance translocation of intra-cellular SphK1 to the membrane. As a consequence SphK1 demonstrated an increase in catalytic activity and bioactive S1P, proposing that the pro-cancer effect, or SphK1-oncogenic ‘inside-outside’ signalling, was reliant on both Ser225-specific phosphorylation and translocation to the membrane [237]. SphK1/S1P extracellular activity has also been shown to promote oncogenicity through initiation of the formation of new vasculature to support new tumor growth. Blocking SphK1/S1P inhibited the formation of new vasculature and hence deprived the tumor of important nutrients [19, 97, 238].

New insights into the structure and function of SphK1 relating to its enzymic role in controlling fundamental cellular processes has been recently reviewed by Adam, Pyne and Pyne [48]. Although the crystal structure of SphK1 is not yet available, comparative analysis of closely related prokaryotic lipid kinase crystal structures identified a potential dimerization region in SphK1, which would confer structural plasticity, or conformational mobility, through phosphorylation and interaction with other proteins and phospholipids. Physiology of enzymatic regulation may well be dependent on the structural plasticity of SphK1 and interactors and dysregulation of these interactions may contribute to disease. There is still a long way to go to understanding the structure and function of SphK1, and knowledge of the SphK1 crystal structure will allow the discovery of druggable allosteric sites and development of specific ligands to modulate SphK1 activity [48].

### A role for SphK2/S1P in oncogenesis

Albeit historically SphK1 is the isozyme associated with oncogenesis, it is important to note that there is new evidence to strongly suggest that SphK2 is overexpressed in many human cancers [239] and dual pro- and anti- apoptotic functions of SphK2 may well be dictated by the cellular milieu within which the protein exists. Pitson and colleagues demonstrated that a relatively low increase in SphK2 levels (2.5 fold), compared to the levels in corresponding normal tissue, could potentially promote cell proliferation and neoplastic transformation, whereby in contrast, high levels of SphK2 are associated with pro-apoptotic signalling, predicted to be through the unique BH3 pro-apoptotic domain, that dominates over the proliferative SphK2/S1P response [239]. On the other hand, Xia and colleagues showed that insulin stimulated phosphorylation of SphK2 in a similar time- and dose-dependent manner compared to SphK1, led to similar activation of cell cycle progression and proliferation in MCF- 7 cells [229].

The SphK2 isozyme contains nuclear import and export sequences, allowing shuttling between the nucleus and cytoplasm [240]. In the nucleus SphK2 complexes with histone H3 and histone deacetylase 1 and 2 (HDAC 1/2) producing S1P that regulates histone acetylation, as part of gene regulation [241]. SphK2-derived S1P also binds hTERT to allosterically mimic phosphorylation and maintain telomere integrity and stability through limiting proteasome degradation [242]. As telomere stabilization is enhanced in cancer S1P stabilization of telomeres potentially enhances tumor growth, however this is somewhat controversial to its role in apoptosis or cell protection against cancer [243]. In addition, FTY720, a competitive inhibitor of SphK2, has been shown to inhibit DNA synthesis and as a consequence, inhibited S1P-stimulated actin rearrangement and reorganized the focal adhesion assembly in MCF-7 cells, potentially regulating the transition of MCF-7 cells from a stationary phenotype to a migratory phenotype [244].

Another example of SphK2 isozyme location influencing S1P function comes from Strub and colleagues who demonstrated S1P, produced by SphK2 in the mitochondria, specifically interacted with prohibitin 2 to control mitochondria respiration [37]. As genetic alteration and aberrant metabolism are two of the hallmarks of cancer, the link between overexpression of SphK2/S1P in the nucleus, affecting genetic gene regulation and stability, and SphK2/S1P altering mitochondria metabolism, may have consequences for cancer progression.

In other studies using RNA interference to selectively ablate each of the SphK isozymes, SphK1 ablation showed no effect on mRNA and protein levels of SphK2 however reduced intracellular S1P levels and increased ceramide (pro-apoptotic lipid), alternatively, SphK2 ablation increased intracellular activity of SphK1/S1P [220]. Anti-intuitively, despite increased S1P levels in these experiments, reduction of SphK2 suppressed proliferation and migratory potential of cells more effectively than SphK1 ablation indicating targeting SphK2 may have stronger anti-cancer effects in cancer therapy [220].

As mentioned previously, there are inherent differences in the composition of the ATP and catalytic binding pockets of the two SphK isozymes, and specific SphK1 and SphK2 compounds have been developed targeting this peptide region to inhibit the catalytic activity of either isozyme and these are listed and referenced in Table 2. SphK2 specific inhibitors, such as the immunosuppressant drug ABC294640 and FTY720 have been shown to have greater or similar anticancer effects than compounds that target SphK1, such as CB5468139, or that target both isozymes, SK-II and ABC294735, making SphK2 inhibitors attractive compounds for anti-cancer therapy [47]. The drug ABC294640 has been shown to reduce the growth of hormone resistant prostate cancer cells [245] and colorectal and colon cancer cell growth [246, 247]. Though SphK1 does phosphorylate FTY720, which acts as a pseudo ligand for S1P, binding of SphK2 is more potent having a 30-fold more ligand-binding efficiency in humans and murine species [248]. The effect of FTY720 binding to SphK2 in the nucleus and preventing HDAC 1/2 repressor gene regulation has also been shown to restore ERα expression and hence restore tamoxifen sensitivity [249].

**Table 2:** SphK inhibitors

SphK inhibitor	SphK selectivity	References
SKi (2-(p-hydroxyanilino)- 4-(p-chlorophenyl)thiazole) or SK1-II	SphK1 and SphK2	[18, 47, 239]
Safingol	SphK1 and SphK2	[283]
L-threo-dihydrosphingosine (DHS)	SphK1 and SphK2	[284]
N,N-dimethyl-D-erythro-sphingosine (DMS)	SphK1 and SphK2	[18]
B‐5354c, F-12509A (Natural products)	SphK1 and SphK2	[18]
ABC294735	SphK1 and SphK2	[47]
Amgen 82	SphK1 and SphK2	[276]
Amidine-based range of sphingosine analogues	SphK1 and SphK2	[18]
MP-A08	SphK1 and SphK2	[271]
ST-1083	SphK1 and SphK2	[285]
S-15183a and S-15183b (Natural product)	Not specified	[18]
PF-543 ((R)-(1-(4-((3-methyl-5-(phenylsulfonylmethyl)phenoxy) methyl)benzyl)pyrrolidin-2-yl)methanol), SK1-5c (CAY10621), SK1-178, VPC96091 (36a), CB5468139	SphK1 SphK1	[111, 286] [287] [239, 288] [239, 288]
SKI-I	SphK1	[97, 289, 290]
LCL351	SphK1	[291]
Compound inhibitors 51 and 54	SphK1	[271, 292]
Balanocarpol	SphK1	[293]
VPC94075	SphK1	[294]
1-deoxysphinganines 55-21 and 77-7 (induces proteasomal degradation -SK1)	SphK1	[295] (55-21)
RB-005	SphK1	[296]
(S)-FTY720 vinylphosphonate	SphK1	[297]
Genzyme	SphK1	[27, 276]
ABC294640	SphK2	[18, 298]
SG-12 and SG14 (sphingosine analog)	SphK2	[299]
SLC5111312 and SLM6041434	SphK2	[300]
F02 thiourea adduct of sphinganine	SphK2	[295]
VT-ME6	SphK2	[301]
(2S,3S,4R)-Pachastrissamine	SphK2	[302]
Trans-12a and Trans 12b	SphK2	[301]
SLR080811, SLP120701	SphK2	[239]
K145	SphK2	[239]

Adapted from [303]

SphK2 also plays a role in cell migration in a cell specific manner. In the breast cancer cell line MDA-MB-453, cell migration towards the epidermal growth factor (EGF) was observed and this migration was abrogated by SphK2-specific siRNA whereas downregulation of SphK2 in HEK293 had no effect on migration [250]. Although it is not understood why SphK2 involvement in cell migration is cell specific, especially as the majority of endogenous SphK2 was present in the plasma membranes of both HEK293 and MDA-MB-253 cell lines and EGF did not affect obvious localization changes in SphK2, it may still be possible that SphK2 may relocate to specialized plasma membrane compartments and produce S1P in the vicinity of its receptors. Also there is some *in vitro* data to suggest that SphK2 can compromise the integrity of the endothelial cell monolayer [251], which may contribute to invasion and migration of cancer cells. The complex interaction between SphK and S1PR with reference to the potential role of SphK2 in regulating metastasis is discussed in more detail in the following section.

As there is indisputable evidence to support SphK2 compensatory mechanisms in the absence of SphK1 in ‘normal’ physiology (at least in mouse models) [19], and the discovery of SphK2 specific inhibitors (FTY720 and ABC294640), acting as competitive inhibitors of SphK2 (not SphK1), with the ability to be phosphorylated by SphK2 and be released from cells to act on S1P receptors, also suggests a wider functionality for this isozyme and its role in neoplasia and cancer [252, 253].

The growing evidence supporting a role for SphK2 in cancer development provides sufficient precedent to warrant the inclusion of SphK2 as a candidate therapeutic target for many types of cancer.

## S1PR EXPRESSION AND MALIGNANCY

There is ample evidence in the literature to suggest that the differential and overexpression of S1PR_1-5_ are involved in cancer progression and metastasis [123, 139, 250, 254–256]. Differential expression of the S1PRs is known to occur at different stages of development, S1PR_1_ expression has been shown to be essential for embryonic development and normal physiological functions, especially in vascular maturation [34, 257], whereas S1PR_2_ and S1PR_3_ were reported to be redundant in this process [42]. In estrogen receptor positive (ER+) tumors high S1PR_1_ and S1PR_3_ were reported to be causally associated with tamoxifen resistance and poor prognosis [92], and *in vitro*, in ER+ MCF-7 breast cancer cells, the SphK1/S1PR_3_ loop promoted breast cancer progression and migration [94]. S1PR/S1P has been shown to be important for osteoclast formation, bone cells that break down bone tissue. A few recent studies have demonstrated that FTY720 binding to S1PR_1_ and or S1PR_2_ prevents osteoclast formation [258, 259]. By blocking osteoclast formation and reducing bone loss, FTY720 may indirectly have added benefits for advanced cancers that migrate to the bone and where metastasis involves considerable osteolysis [260].

**Figure 3:**
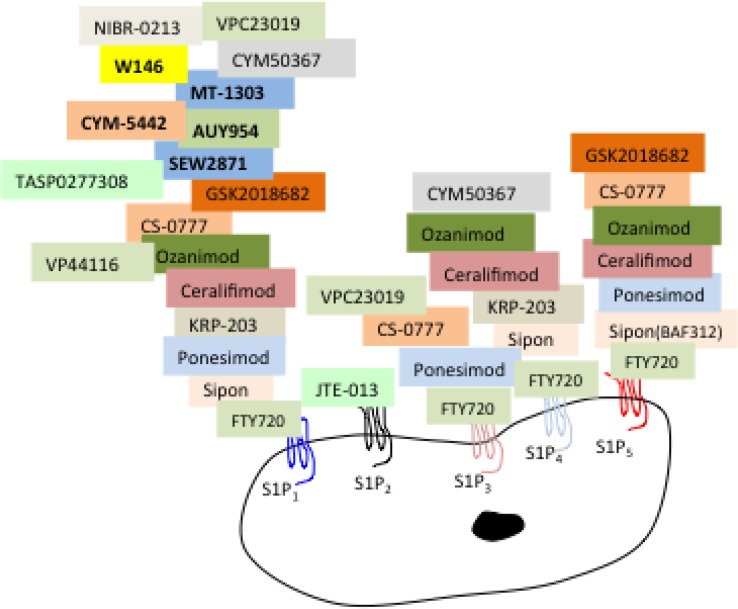
Designer anti-S1PR1-5 drug specificity Update of 1^st^ and 2^nd^ generation S1P receptor modulators developed to target individual and multiple S1P receptors. Each of the receptor modulators binds to individual or multiple receptors to block or activate the SIPRs as illustrated. MOST S1PR modulators target 2 or more receptors. Sipon (BAF312) - Siponimod is a S1P1 = 5 > 4 modulator; Ponesimod (ACT-128800) is an agonist for S1P1 > 5 > 3; KRP-203 is an agonist for S1P1 > 4; Ceralifimod (ONO-4641) is an agonist for S1P1 = 5 > 4; Ozanimod (RPC1063) is an agonist for S1P1 > 5 > 4; Cs-0777 is an agonist for S1P1 > 5 > 3; GSK2018682 is an agonist for S1P1 > 5. JTE-013 is a competitive antagonist specifically for S1P2. CYM50367 targets S1PR1/4 and VPC23019 targets S1PR1/3. Sew2871, AUY954, MT-1303, CYM-5442, W146, NIBR-0213, TAS0277308, are listed as selective S1P1 modulators. These novel S1P receptors and downstream signaling pathways and functions are reviewed in [15, 258, 281].

Interesting, in many cancer cells a shift in S1PR expression is observed whereby, S1PR_1_ expression is lost or decreased and S1PR_2_ and/or S1PR_3_ are predominantly expressed [123, 139, 254, 255]. Moreover, within cancer cell sub-types, differences in S1PR expression correlate with the metastatic or non-metastatic cancer phenotype [123, 139]. Yamashita and colleagues demonstrated that gastric cells expressing high levels of S1PR_3_ and low levels of S1PR_2_ were causally correlated with the migratory phenotype in response to binding S1P, and in the same study, gastric cells expressing predominantly S1PR_2_ did not have the same chemotactic responses to S1P and did not migrate [123]. Specific inhibition of the S1PR_2_ receptor by JTE-013 (refer to Figure 3) abolished the inhibitory effects of S1P in S1PR_2_ expressing gastric cells. Similarly, in mouse melanoma cells overexpression of S1PR_2_ has been demonstrated to abolish the metastatic potential whereas S1PR_1_ aggravated metastasis [139]. Conversely, SphK1/S1P elevation of S1PR_2_ was shown to be associated with increased metastatic potential in the lung, breast [261] and brain [262], which is seemingly contradictory. A twist in the story and a clue to the seeming contradiction was the discovery of high S1PR_4_ causal association with recurrence, reduced survival rates and metastasis in estrogen receptor negative breast tumors [256]. This led to the further finding that SphK2/S1PR_4_ prevented nuclear translocation of S1PR_2_, which in turn, prevented tumor growth, providing support for sub-cellular distribution of S1PR_2_, which is important for oncogenic suppression [263]. Thus, these findings may partly explain the contradictory S1PR_2_ subtype-specific regulation of metastasis, suggesting SpK/S1P stimulated cell migration and proliferation is dependent on the cell-specific diversity of the SphK and S1PR_1-5_ expression [123, 256, 261, 263, 264]. The S1PR sub-type, the level and the localization of S1PR expression is critical for the degree of cancer progression and contributes to the increasing complexity and challenges for SphK andS1PRS as oncotargets.

## SPHK IN CANCER DRUG RESISTANCE

It comes as no surprise that the SphK pathway is associated with anti-cancer drug resistance given the overexpression and aberrant expression of SphKs, S1P, and S1PRs lead to cancer development and metastasis. Overexpression of SphKs, S1P and the S1PRs are causally associated with cancer drug resistance to conventional cancer treatments (radiotherapy, chemo- and hormone therapies) in many types of cancers, including breast [92, 230], pancreatic [265], and prostate cancers [266]. Cancer patients overexpressing SphK1 tend to have poorer survival rates [129, 223]. As discussed in this review, cancer cells can become dependent on the SphK1 enzyme for survival and proliferation, termed non-oncogenic addiction. In estrogen receptor (ER) positive breast cancer cells overexpression of SphK1 increases tamoxifen resistance and ablation of SphK1 restores tamoxifen sensitivity [92, 230]. S1P binding to S1PR_3_ is associated with SphK1 translocation of SphK1 to the plasma membrane to promote tumorigenesis in ER+ breast cancer [94]. In ER negative breast cancer high tumor levels of S1PR_4_ and SphK1 are associated with poor patient prognosis [256, 267], and in HER2+ cells S1PR_4_/HER2+ signaling is associated with metastasis [267]. Similarly overexpression of SphK1 correlates with androgen independent refractory cells [105, 268].

The mechanism for SphK-induced drug resistance is unclear, however, much effort has gone into developing anti-SphK designer drugs to overcome drug resistance as discussed in greater detail in the next section. Nonetheless, what is emerging is the complexity of SphKs isozyme and isoform expression pattern, forming the basis of this review, and how aberrant expression of these enzymes is posing problems in the design and implementation of anti-SphK drugs in the clinic. In particular we review how aberrant expression of the hSphK1 isoforms can confer resistance to SphK1 targeted drugs [25, 26, 102] highlighting the challenges faced in anti-SphK drug treatment in cancer therapy.

## SPHK/S1P DESIGNER DRUGS

Since SphKs have been recognized as major driver proteins in many cancer types, there has been much interest in targeting the overexpression of human SphK1 (hSphK1)/S1P as an anticancer treatment [11, 15, 27, 48, 57, 60, 239, 269–276]. However, the design of drugs to inhibit SphK/S1P action has inherent complexities due to the diversity of SphK functions and its important role(s) in normal physiology. Thus pan-SphK is not considered to be an ideal solution for cancer therapeutic targeting. Current strategies in development for cancer treatment are targeted towards designer drugs to prevent overactivity of S1P action either by selectively targeting and blocking S1P receptors [15, 78, 271, 277–279] or targeting the S1P producers, SphK1 and/or SphK2 [27, 108, 275, 280]. Inhibitors of individual or combinations of S1PR_1-5_, to block specific S1PR signaling (Figure 3, Table 3), or block SphK1 and/or SphK2 catalyst activity are available and listed in Table 2 (adapted from [15, 271, 281]). There are a number of specific and non-specific S1PR modulators currently in clinical use and clinical trials for diseases such as multiple sclerosis (primary progress and relapse remitting), autoimmune disease, inflammatory disorders, kidney transplantation rejection, liver and renal disorders, and acute stroke, and these have recently been reviewed [281]. Considering anti-SphK drugs such as the immunosuppressor prodrug FTY720 are being successfully and safely used in the clinic, adds to their appeal as anti-cancer therapy. In particular, FTY720 binding to SphK2 in the nucleus has been shown to inhibit the SphK2/histone H3/HDAC 1/2 repressor complex therefore allowing the transcription of proteins, such as p21, which mediate cell cycle arrest [249].

**Table 3:** Comparative selectively of the S1P modulators

S1P modulator	S1PR selectivity	References
**Agonists**		
FTY720*	S1P_1_>S1P_5_>S1P_4_>S1P_3_	[110]
Fingolimod* and phosphorylated fingolimod (Trade name: Gilenya)	S1PR_1_, S1PR_3_, S1PR_4_, S1PR_5_	[234, 304–308]
S1P-specific antibody	Depletion of S1P	[18]
CS-0777*	S1P_1_>S1P_5_>S1P_3_	[309]
Ponesimod (ACT-128800)	S1P_1_>S1P_5_>S1P_3_	[271]
RPC0163*	S1P_1_>S1P_5_>S1P_3_>S1P_2_	[271]
ONO-4641	S1P_1_>S1P_5_>S1P_4_	[310, 311]
Siponimod (BAF312)	S1P_1_>S1P_5_>S1P_4_	[312]
GSK2018682	S1P_1_>S1P_5_	[271]
SEW2871	S1P_1_	[313, 314]
AUY954	S1P_1_	[315, 316]
MT-1303	S1P_1_	[271]
KRP-203 and phosphorylated KRP-203	S1P_1_>S1P_4_	[317, 318]
AAL(R) and phosphorylated AAL(R) (FTY720 analogue)	S1PR1, S1PR3, S1PR4, S1PR5	[294, 319, 320]
CYM-5442	S1P_1_	[321, 322]
VPC23153	S1P_4_	[323, 324]
W-061	S1P_1_> S1P_5_> S1P_4_> S1P_3_	[310, 325]
CYM-5442	S1P_1_	[321, 322]
CYM-5478*	S1P_2_	[326]
**Antagonists**		
VPC44116 VPC23019 VPC25239	S1P_1_ and/or S1P_3_	[18] [327]
TASP0277308	S1P_1_	[328]
W146	S1P_1_	[329, 330]
JTE-013	S1P_2_	[189, 331]
NIBR-0213	S1P_1_	[329]

Note: *S1P modulators can act as functional antagonists.

More detailed reviews [15, 18, 56, 57, 332].

A number of selective sphingosine-based inhibitors and allosteric activators of SphK have been designed to combat the hyper-proliferative signaling of cancer and other diseases and the value of these compounds are reflected in the many filed patents including: Selective inhibitors and allosteric activators of sphingosine kinase, patent - WO 2014118556 A2; and amidine analogs that can inhibit activity of SphK1 and SphK2 patent - WO 2009146112 A1; long chain base sphingosine kinase inhibitors targeting hSphK1 and 2: WO 2013119946 A1, reviewed in [282].

### Inherent problems with SphK/S1P/S1PR designer drugs

Targeting SphKs or specific or combinational S1PRs for cancer therapy nonetheless has inherent problems. Many of the earlier S1P and SphK inhibitors were problematic exhibiting non-specific and off-target effects and with low potency. As discussed above, individual cancer cells express different S1PRs sub-types, and these sub-types are associated with cancer development and malignancy [123, 139]. For example, as schematically shown in Figure 2, most of the S1PR modulators target S1PR_1_ however S1PR_1_ may not be expressed or be reduced in cancer cells. Furthermore, the specific interactors of the S1PRs and the location of expression of the S1PRs may dictate the cell's propensity to become malignant. Until recently SphK1 has been purported as the main driver of non-oncogenic addiction and proliferation. Albeit, recently developed compounds such as PF543, which inhibits hSphK1 with a greater potency (> 100-fold) than for hSphK2, and the dual inhibitor Amgen 82, which is proving to be the most potent dual inhibitor available, reduce S1P production, but do not induce cytotoxicity in cancer cells at pharmaceutically relevant concentrations, undermining their clinical application in cancer therapy [27, 276]. As shown in 1483 head and neck cancer cell lines tested PF543 failed to increase ceramide, which is essential for apoptosis induction [286]. Another perceived disadvantage of the SphK1 inhibitor PF543 was discovered when it was administered to mice and exacerbated an inflammatory response [56].

Focus is now drawn to inhibition of hSphK2 and the most studied is the specific SphK2 inhibitor, ABC294640 compound [282], now in phase I/II clinical trials (Table 4). Compound ABC294640 has been shown to effectively suppress proliferation accompanied by a decrease in intracellular S1P levels and an increase in ceramide [46]. Although this drug has shown significant efficacy in cancer cell and animal models, as mentioned previously, the specificity of ABC294640 is questionable. As Pyne and colleagues discovered ABC294640 induced proteosomal degradation of the SphK1a isoform in HEK293T and androgen-independent prostate cancer cell lines showing ABC294640 was not a specific SphK2 target (cited in [56]). Second generation SphK2 inhibitors such as SLR080811 are even more potent, however, anti-intuitively, SLR080811 inhibition of SphK2, increases circulating S1P levels significantly, which has potential negative consequences as S1P has been shown to drive angiogenesis [46, 282].

Despite the many drug compounds developed to target the SphK pathway, anti-SphK drug design is still a challenge due to the functional plasticity of the SphK proteins which are activated by phosphorylation, protein interactions and the suggestion that their catalytic function and regulation may be dependent on conformational mobility as reviewed [48]. Also, due to the similarities and overlap in the lipid binding pockets of the enzymes in the sphingolipid metabolic pathway, achieving high specificity is difficult [48].

As will be discussed, aberrant expression of SphK1 isoforms can also affect the efficacy of current cancer therapies [25, 26] and may have consequences for anti-SphK1 and S1P therapy in the presence of comorbid conditions [15, 28]. Although progression has been made towards understanding the SphK/S1P/S1PR pathways, this has also opened a Pandora's box. As listed in Tables 2 and 3 a plethora of SphK/S1P/S1PR modulators have been developed. Optimistically, a few of these SphK modulators are now in Phase I and II clinical trial for cancer treatment (Table 4), nonetheless very few of these modulators to-date have been approved for clinical use for cancer therapy.

**Table 4:** SphK/S1p/S1PR drugs in clinical trial

Drug	SphK selectivity	Indications	ClinicalTrials.gov identifier	Phase
Safingol	Sphingosine derivative, PKC inhibitor	Solid tumors, combined with fenretinide Solid tumors, combined with cisplatin	NCT01553071 NCT00084812	I [283] I (Completed)
ABC294640	SPHK2 inhibitor	Pancreatic cancer Diffuse Large B Cell Lymphoma Multiple Myeloma Carcinoma, Hepatocellular	NCT01488513 NCT02229981 NCT02757326 NCT02939807	I I Not yet recruiting. Not Yet recruiting.
Sonepcizumab (ASONEP)	S1P-specific monoclonal antibody	Solid tumors Unresectable and Refractory Renal Cell Carcinoma	NCT00661414 NCT01762033	I (Completed) II (Terminated)

Adapted from [78]

## DISCOVERY OF SPHK1 ISOFORMS IN MOUSE AND HUMANS

There is undisputable evidence to support a significant role for aberrant SphK1 and SphK2 isozyme expression in cancer development. Despite a huge body of basic research and studies on the biochemical regulation of the SphK pathways, there has been a delay in the introduction of drugs that target SphK function for cancer therapy into the clinic. There is now supporting evidence to suggest that aberrant expression and/or inappropriate cellular localization of SphK1 and SphK2 isoforms contribute to both oncogenicity and anti-cancer treatment resistance. The following sections review the characterization of the SphK isoforms and their potential oncogenic role and prognostic significance of SphK1 and SphK2 isoform expression.

### Characterization of the mouse SphK1 isoforms

**Figure 4:**
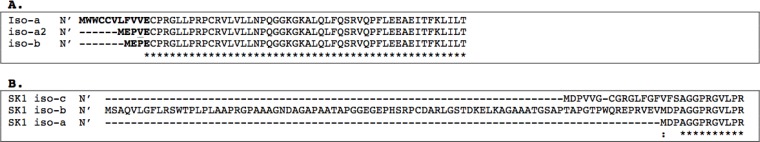
Alignment of the N-terminal amino acid sequences of the mSphK1 and hSphK1 isoforms **A**. Three mSphK1 isoforms (iso-a, iso-a2, and iso-b) have been identified to date. Iso-a has an additional 6 and 7 amino acids at the N-terminus (N’) compared to the mSphK1a2 and mSphK1b isoforms respectively. There is 10 amino acid overall sequence dissimilarity when comparing mSphK1a to mSphK1a2 and mSphK1b. MSphK1a2 differs from mSphK1b by an insertion of a valine (V) in the N’ changing the sequence MEPVE (iso-2a) to MEPEE (iso-b). **B**. Human SphK1 is expressed as 3 isoforms (a, b, and c) which have identical in sequences except for the N-terminus (N’). The SphK1b N’ has an additional 86 amino acids upstream of the Met start codon of SphK1a, as illustrated. SphK1c has an additional 14 amino acids upstream from the SphK1a start codon, which translates to a difference of 17 amino acids between the SphK1a isoform compared to SphK1c. Legend: * = complete amino acid sequence identity across all isoforms; : = similarity of amino acids across 2 isoforms. Sequences aligned using CLUSTAL Omega (1.2.3) multiple sequence alignment tool.

SphK1 and SphK2 isozymes are both expressed as variant isoforms in mice and humans. Murine SphK1 (mSphK1) was the first mammalian SphK to be cloned and shares amino acid sequence homology with the corresponding *Caenorhabditis elegans (c. elegans)*, and yeast counterparts [23]. Much of the early characterization of the mammalian SphK was derived from the mouse model. MSphK1 is ubiquitously expressed in many different mouse tissues but at differing levels, depending on the tissue type [23, 333]. Two similar mSphK1 isoforms, mSphK1a and mSphK1b, identified by the Spiegel group, were found to differ only at the N-terminal where the mSphK1b isoform was identified to be seven amino acids longer than the 1a isoform, however showing an actual 10 amino acid difference overall (Figure 4) [23]. Despite the strong sequence similarities the enzymatic traits of the two lipid kinases were found to be very different [334]. Compared to mSphK1a, mSphK1b was shown to differ in cellular location, oligomerization properties and post-translational modifications [334]. SphK1b was observed to be more unstable than the 1a isoform and even though the predicted mass of the b- isoform was 43.3kDa, when run on SDS-PAGE exhibited an unusually fast mobility and the band was detected at 34kDa [334]. This suggested that the native conformational structures of the two isoforms were vastly different, and the b-isoform has an SDS-resistant structure. Although the 3 dimensional structures of the mSphK isoforms have not been characterized, the catalytic domain is adjacent to the N-terminus and the proposed conformational differences between the isoforms are likely to contribute to the differences in enzymatic traits. Although there are only few differences in the amino acid sequences of mSPHK1a and mSPHK1b [333], the mSphK1a isoforms exhibit 3-fold greater activity than the mSphK1b isoform. The mSphK1b isoform, which is located predominantly on the plasma membrane, was found to be more unstable compared to the 1a-isoform and did not accumulate in the cell [334]. Treatment with the proteasome inhibitor MG132 slightly increased the abundance of the mSphK1a isoform whereas the effect on mSphK1b abundance was significantly increased, suggesting involvement of the proteasome in the differential degradation of the two mSphK1 isoforms [334]. Site-directed mutagenesis of unique Cys residues in the mSphK1b isoform led to an increase in mSphK1b activity and prevented membranous degradation of mSphK1b upon addition of proteasome inhibitors [334]. Interestingly, mSphK1a was more susceptible to degradation when membrane bound suggesting it was the ability of isoform binding to the membrane that was responsible for mSphK1 protein stability [334]. Structural differences were also observed between the -1a and -1b isoforms whereby mSphK1b was mainly found as homo-oligomers and mSphK1a isoforms were mainly observed as monomers, allowing a greater diversity of cellular function [334].

**Figure 5:**
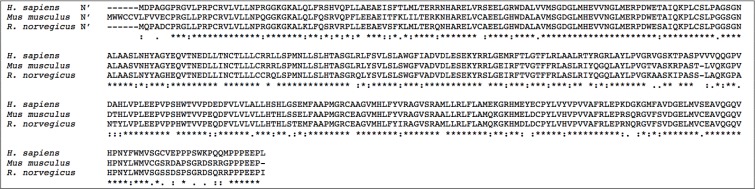
Comparative alignment of human and murine SphK1 amino acid sequences SphK1 amino acid sequence alignment showing similarities and differences in SphK1 sequences across human (H. sapiens), mouse (Mus musculus) and rat (R. norvegicus) species. Legend: * = complete amino acid sequence identity across all three species; : = similarity of amino acids across 2 species. Sequences aligned using CLUSTAL Omega (1.2.3) multiple sequence alignment tool.

It was only in 2006 that a third murine mSphK1 isoform, mSphK1a2, was reported [334]. In comparison to mSphK1a, the SphK1a2 isoform contains an extra valine (Val) residue due to an insertion of three base pairs in the coding- region. Thus mSphK1a contains the sequence Met-Glu-Pro whereas the mSPHK1a2 isoform has the sequence is Met-Glu-Pro-Val revealing a greater similarity between mSphK1a2 and mSphK1b (Figure 4a). The mSphK1a2 isoform had very similar stability and function to its mSphK2 counterpart and was the most ubiquitously expressed, followed by mSphK1a and mSphK1b in decreasing order of abundance [334]. Much of our biological characterization of the SphK isoforms is derived from murine models however, although there is high amino acid sequence homology between the human and mouse SphK proteins at the C-terminal, there are vast differences in the N-terminal sequences and apparent differences in the ligand binding pockets between the different SphK1 isoforms of mammalian species (Figure 4). Thus, while great inroads to SphK function have been acquired through studying the murine models, the functionality of hSphK isoforms cannot directly be compared to mSphK isoforms due to their profound differences at the N-terminal [271]. Here we focus on what is known about the human SphK (hSphK) isoforms and the further challenges associated with targeting SphK/S1P as a therapeutic target for multiple diseases.

### Characterization of human SphK1 (hSphK1) isoforms

A putative hSphK1 was predicted in 1998 sharing sequence homology with *c. elegans*, yeast and murine SphK1 sequences [23], however the hSphK1 was not sequenced and characterized until 2000 [206, 335]. Due to the high homology in the hSphK1 C-terminal of all the mammalian species sequenced, hSphK1 could be identified immunohistochemically in human tissue using a rabbit polyclonal antibody, thus confirming the diversity of hSphK1 expression in different tissue types [333]. Using Northern blot analysis, hSphK1 was observed to be widely expressed in human tissues with highest expression levels in adult liver, kidney, heart and skeletal muscle [335]. Since the first discovery of human SphK1, three unique isoforms of the hSphK1 have been identified to date varying only at the N-terminus: hSphK1a (NM_001142601, Q9NYA1); hSphK1b (NM_182965: Q9NYA-2) and hSphK1c (NM_021972: Q9AYA1-3), the Genbank^TM^ and Uniprot accession numbers, respectively, have been provided. Both hSphK1a and hSphK1c are similar in size (42.5kDa and 43.9kDa respectively) with the 1c-isoform having an additional 14 amino acids at the N-terminus (Figure 4b). Both hSphK-1a and -1c have similar mobility on SDS PAGE and in some reports have been referred to as hSphK1a and hSphK1a+14 to distinguish between the two isoforms [25]. The hSphK1b (51kDa) isoform contains a unique upstream 86 amino acid sequence at the N-terminal region (Figure 4b). It is noted that in some reports the hSphK1c isoform refers to the longer isoform (470 amino acids) and the hSphKb isoform refers to hSphKc (398 amino acids) [53]. For this review, the convention will refer to hSphK1b as the 51kDa (470 amino acids).

Much of the literature does not specify the human SphK1 isoform used in the reported studies, and most *in vitro* studies have conventionally used the shorter hSphK1a isoform to determine structure and function of hSphK1 in the cell [48]. To-date few reports have defined the distribution of the hSphK1 isoforms in cells, or in different tissue types, and most studies use stable or transient overexpression of the hSphK1 isoforms to define their biological significance. Using overexpression of hSphK1 isoforms in human cells hSphK -1a and -1b isoforms have been demonstrated to have similar S1P activity [25, 28, 53] and both isoforms translocate to the plasma membrane [25]. However, what is emerging is that there are functional differences in the activity of the human SphK1 isoforms. Hla and colleagues provide evidence showing hSphK1a is preferentially secreted from cells and activates extracellular S1P/S1PR_1_, whereas hSphK1b and hSphK1c are more likely to be retained in the plasma cell membrane [53]. The suggestion is that hSphKa may contribute to the establishment of the vascular S1P gradient and vascular integrity under normal physiological conditions. As the SphK rheostat is involved in inflammation [336], it is possible that hSphK1a has a distinct role in inflammatory disease.

### Common and specific interactions of human SphK1 isoforms

Studies using the hSphK1b isoform suggest that the extra 86 amino acids at the N-terminal may contribute to conformational changes relevant to the diversity of SphK1 function and may impact on efficacy of hSphK1 directed drug administration and outcome [15, 57, 271]. As far as we are aware, there is only one study to-date that used a multiplex-based stable isotope labeling with amino acids in cell culture (SILAC) co-immunoprecipitation to identify unique and common interacting partners of the two major hSphK isoforms (1a and 1b) elucidating possible ways by which these interactions may influence cancer pathophysiology [28]. Gene ontology analysis of the functional roles for the interacting partners of the two isoforms showed strong similarities demonstrating common functions as expected. Of major interest is the difference in the interactions conferred by the unique extra 86 amino acids of the hSphK1b, which not only allows specific binding to the unique upstream N-terminal region but also confers conformational differences between the two isoforms affecting specific binding affinity. The PP2A serine/threonine phosphatase, one of the most well characterized hSphK1 partners responsible for deactivating SphK bound with greater affinity to the hSphKa (43kDa) isoform. As expected, in this study, most of the isoform specific interactions were associated with the hSphK1b isoform. These interactions included various proteasomal sub-units, and latent-transforming growth factor β-binding protein 1. The unique putative proteasomal binding sites suggest that this isoform may be more susceptible to proteasomal degradation in a breast cancer cell line. This observation was supported by a prominent increase in the hSphK1b isoform upon treatment with the proteasomal inhibitor MG132 whereas hSphK1a protein levels showed little change post treatment indicating hSphK1b has a higher protein turnover than hSphK1a in cells [28].

Although specific functions for each of the SphK1 isoforms have yet to be identified *in vitro* and *in vivo* we have some evidence to suggest unravelling the individual and common or overlapping functions SphK1 isoforms will add further complexity to targeting SphK in cancer treatment. A full list of the reported SphK1a (43kDa) and SphK1b (51kDa) interactors can be found in [28]. In these studies by McGowan and colleagues the SphK1a (51kDa) and the SphK1b (43kDa) isoforms were independently, stably overexpressed. To-date, no studies have looked at the likelihood and potential effects of hSphK1 heterodimerization (hSphK1a-hSphK1b) and their interactors. These types of dimerization studies would add to our knowledge of the structural plasticity of SphK1, add to the repertoire of protein-interactions, and increase our understanding of the extensive downstream functions and cellular effects of SphK1 isoforms. As recently highlighted by Adams, Pyne and Pyne [48] the catalytic function and enzyme regulation through SphK1 is likely to be dependent on conformational mobility and an understanding the dynamic changes in hSphK1 structure will provide more avenues for the development of SphK modulators in targeted cancer therapy.

### hSphK1b isoform - a link between diabetes and cancer treatments

An important hSphK1b-specific interactor identified was the dipeptidyl peptidase 2 (DPP2), which is involved in the regulation of glucose metabolism [28]. DPP2/4 inhibitors block degradation of hSphK1b but have no effect on the hSphK1a isoform. As DPP inhibitors are used in diabetic treatment, these treatments would increase the propensity towards elevated hSphK1b and S1P activity. The suggestion that DPP inhibitors may increase cancer risk and increased S1P activity has been reported as a mediator of oncogenicity: highlighting the “ying and yang” of comorbidity treatments (reviewed in [15]).

### hSphK1 isoform specific anti-SphK drug efficacy

Previous studies have shown the involvement of the proteasome in the regulation of hSphK1, where novel inhibitors of SphK1-S1P activity induced the proteasomal degradation of this protein in prostate cancer cell lines [266]. Androgen-independent prostate cancer cell lines demonstrate higher levels of SphK- 1a and 1b than androgen sensitive prostate cells [25, 266]. Further studies demonstrated proteasome regulation and degradation of hSphK1 was isoform dependent and cell-type specific [25, 26, 102]. These studies clearly show that cellular phenotypic changes in cell lines also affected proteasomal degradation and stabilization of the two major hSphK isoforms, which in turn affect anti-SphK drug efficacy. As demonstrated in prostate cancer cell lines, hSphK1b was less sensitive to proteasomal degradation in androgen-independent LNCaP-A1 cells compared to LNCaP androgen-sensitive cells [25, 26]. The inhibitor of the SphK1 and SphK2 isozymes, SKi (2-(p-hydroxyanilino)-4-(p-chlorophenyl) thiazole)) induced proteosomal degradation of hSphK1a and hSphK1b in androgen-sensitive prostate cancer cells, however failed to promote degradation of hSphK1b in androgen-independent prostate cancer cells. The apparent inability of the Ski inhibitor to affect the hSphK1b expression in androgen-independent cells was found to be due to a compensatory increase in hSphK1b mRNA expression [25].

Indeed, further studies demonstrated that the inhibitor of SphK2, ABC294640 (3-(4-chlorophenyl)-adamantane-1-carboxylic acid (pyridin- 4-ylmethyl)amide) was able to reduce the expression of SphK1a in androgen-independent LNCaP-AI cells and this was prevented by pre-treating these cells with the proteasomal inhibitor, MG132 [102]. Overall the major findings from these challenging studies are 1) the ubiquitin-proteasomal degradation of hSphK1 is important in cell survival, 2) differential proteasomal degradation of hSphK1a and hSphK1b is cell-phenotype specific, 3) differential regulation of hSphK1a and hSphK1b may contribute to anti-SphK/S1P therapy resistance as demonstrated in prostate cancer cells [25, 26].

Although these studies provide some insight into the distribution of SphK1 in human tissues and the importance of SphK1 in disease, to date there have been no explicit studies on the relative expression levels of the variant SphK1 isoforms in human tissue, providing some open and very pertinent questions that are yet to be answered. The caveat of most *in vitro* studies is the reliance on studies of the overexpression of SphK1 in stable cell lines and may not truly reflect SphK1 isoform expression in normal physiological or tissue samples. It is currently unknown whether one or both isoforms are expressed in normal cells and whether changes in isoform expression levels occur in, or contribute to, disease states (i.e. in normal cells and cancer cells and different disease states). Determining the relative expression levels of each of theSphK1 isoforms may reveal novel roles for the two major SphK1 isoforms in cancer and other disease development and/or progression.

## CHARACTERIZATION OF HSPHK2 ISOFORMS AND THE POTENTIAL OF HSPHK2B AS AN ONCOTARGET

**Figure 6:**
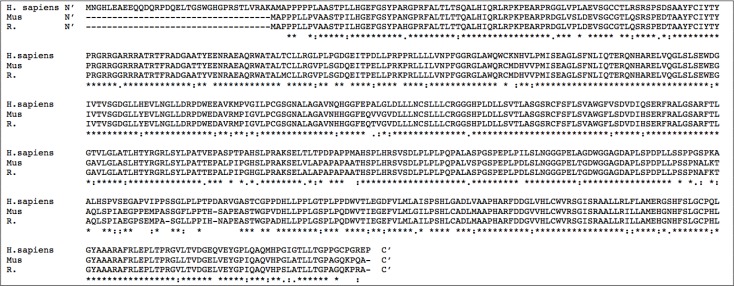
Comparative alignment of human and murine SphK2 amino acid sequences SphK2 sequences have been aligned to show similarities and differences between the SphK2 isozyme sequences across human (H. sapiens), mouse (Mus musculus) and rat (R. norvegicus) species. Legend: * = complete amino acid sequence identity across all three species, : = similarity of amino acids across 2 species. Sequences aligned using CLUSTAL Omega (1.2.3) multiple sequence alignment tool.

The SphK2 isozyme is gaining much prominence as a cancer therapeutic target and may prove to have greater efficacy as an oncotarget than its comparative isozyme hSphK1, which is driving the development of the hSphK2 inhibitors (Table 2), and the ongoing clinical trials of the hSphK2 ABC294640 (Table 4). However, we know little about the structure and function of SphK2 and even less regarding the hSphK2 isoforms. The SphK2a genes were cloned in both murine and humans in 2000 and the comparison is shown in Figure 6 [16]. Interestingly, the SphK2b isoform is not expressed in the mouse therefore functional isoform-specific studies have been limited to cell lines [337]. The SphK2 encodes at least 4 predicted variants [a-d] however 5 isoforms have been annotated: isoform 1 ‘a’ (NM_020126; Q9NRA0-1); isoform 2 ‘b’ (NM_001204158.2; Q9NRA0-2); isoform 3 ‘a’ (NM_001204159.2; Q9NRA0-3); isoform 4 ‘c’ (NM_001204160.2; Q9NRA0-4); and isoform 5 ‘d’ (NM_001243876.1; Q9NRA0-5); the Genbank^TM^ and the Uniprot accession numbers, respectively, are provided. *Note: transcripts for isoform 1 and 3 encode the same isoform ‘a’*. Comparison of SphK2a-d is illustrated in Figure 6.

**Figure 7:**
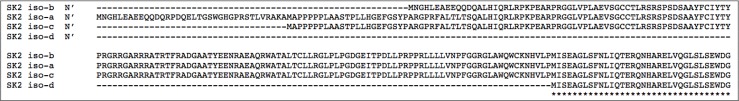
Predicted N-terminal sequence alignment of the hSphK2 isoforms hSphK2 has 5 predicted isoforms differing only at the N-terminus as shown (iso-a, iso-b, iso-c and iso-d). The SphK2c isoform has a predicted 59 amino acid insertion in the N-terminal region compared to SphK2b. SphK2c has a predicted Met start site that is 36 amino acids downstream of the SphK2a (iso-a) start codon. SphK2d has the shortest sequence with a truncation of 86 amino acids from the SphK2a start codon. Legend: * = complete amino acid sequence identity across all isoforms. Sequences aligned using CLUSTAL Omega (1.2.3) multiple sequence alignment tool.

The hSphK2a and -b isoforms have been confirmed with the b-isoform containing 36 extra amino acids at the N-terminal, the c and d isoforms are predicted [46]. The hSphK2-a, b and c isoforms contain five conserved catalytic regions (nominated C1-C5) whereas SphK2d does not possess two of these catalytic regions (C4-C5) and therefore is most probably inactive [46]. Original studies in human cell lines refer to the longer isoform (b-isoform), containing the additional 36 amino acids at the N-terminus, as hSphK2-L and the short isoform (a-isoform) as hSphK2-S [337]. Firstly, the longer isoform was found to be predominant in the human cell lines tested and secondly, these studies defined distinct differences in the physiological role of the N-terminal extension of hSphK2. To-date hSphK2 isoforms have been reported to differ in their subcellular localization with isoform 1 ‘a’ located in the cytoplasm and the membrane, and isoform 2 ‘b’ reported to be located at the lysosome membrane {reference: ncbi.nim.nih.gov;gene:56848]. Billich and colleagues presented data showing that hSphK2-L was involved in the regulation of cell proliferation and apoptosis in serum deprivation and this was cell-type dependent [337].

Based on kinetic studies, the N-terminal extension of the human SphK2b (hSphK2-L) has been reported to be responsible for more efficient binding to FTY720, a sphingosine analog, with more effective activation than the shorter isoforms [248]. This observation is interesting due to the use of FTY720 currently in the clinic for multiple sclerosis and its promise in cancer therapy. Hence, SphK2b may prove to be a more effective anti-cancer target *in vivo*. Work to characterize the functions of the known isoforms is an ongoing requirement for our understanding of the cellular roles of these isoforms and their contribution to disease states and the existence of additional SphK2 variants remains a possibility.

## SPHK/S1PRS AS DIAGNOSTIC OR THERAPEUTIC MOLECULAR BIOMARKERS IN CANCER

Given the considerable evidence showing increased SphK1/S1P/S1PRs is present in many types of cancer, as discussed in this review and others [98, 223, 338, 339], there is the potential for SphK isozymes, isoforms and S1P and S1PRs as potential new biomarkers for clinical diagnosis and prognosis, for early cancer detection, as an indicator of progression and tumor aggressiveness. Prime examples include breast, cervical, prostate, head and neck, and brain cancers. Overexpression of SphK1 and differential S1PRs distribution are associated with poor prognosis and disease stage in breast cancer and may prove to be strong candidates as diagnostic and prognositic markers for ER+ and ER- breast cancers, endocrine resistance, and metastasis [11, 230, 340]. In cervical cancer SphK1 was significantly increased in comparison to normal tissues and patients had a higher probability of recurrence and lower overall survival [130]. Likewise overexpression of SphK1 is found in aggressive gliomas and is associated with poor survival [341].

Nevertheless, SphK1 overexpression is not an indicator for all cancers. Measurement of SphK/S1P and S1PR levels as a cancer diagnostic marker has to be carefully considered as biomarkers as overexpression is not necessarily related to poorer prognosis. In prostate cancer decreased levels of circulating S1P was reported during prostate cancer progression potentially stemming from a significant decrease in erythrocyte SphK1 levels, and this S1P decrease correlates with high prostate specific antigen (PSA) levels, lymph node status and may be an early marker for androgen independent tumors [339]. As discussed above, SphK2 is also gaining ground as a marker of cancer progression [107, 220].

In summary, although SphK1 is highly expressed in many cancer cell types, compared to its contemporary cell type, its suitability as a diagnostic or therapeutic biomarker for all cancers is still unknown. For some cell types it may be an indicator of cancer development and of resistance to conventional cancer therapies. As highlighted in this review, aberrant SphK isozyme and isoform expression levels and cellular localization, differential S1PR_1-5_ expression, and intracellular and extracellular S1P levels are all associated with cancer initiation, development, progression and metastasis. Further profiling and characterization of the SphK pathway will provide a more detailed analysis for future diagnosis and prognosis in the various cancer-types. The feasibility and complexity of developing a method to detect SphK/S1P/S1PRs as biomarkers in the clinical samples, such as blood samples, is another consideration in the future.

## KEY ISSUES AND FUTURE CHALLENGES FOR SPHK AS AN ONCOTARGET

Disruption of the SphK rheostat is now well recognized as a hallmark of cancer development and an instigator of cancer treatment resistance. Nonetheless, SphK balance of sphingosine to S1P is necessary for normal cell physiology so ablating the function of SphK has clinical concerns for deleterious off-target effects.

In this review we have provided an overview of the promise, and the problems, of targeting SphKs, S1P and S1PRs as novel cancer treatments and outlined the approach of pharmacologically targeting SphK1 and/or SphK2 to alter their activity so that clinically beneficial changes in cancer patients can be achieved. In the absence of mutations in SphK and S1PRs it has been difficult to determine the extent of their involvement in cancer and non-oncogenic addiction has been proposed as the mechanism driving SphK-associated malignancy. Hence, the term drugging the addict(s) (SphK1, SphK2 and S1PRs) has been the driving force for novel cancer drug design. As listed in Tables 2 and 3 there is no paucity to the growing list of anti-SphK, anti-S1P, and anti-S1PRs modulators, of which some are in clinical trials, Table 4. While SphK/S1PR modulators hold much promise as additional novel cancer therapies, as indicated in this review, there are still many challenges facing anti-SphK drug use in cancer therapy. Although SphK1 has historically been described as a pro-proliferative lipid enzyme, whereas SphK2 has been described for its pro-apoptotic effects, in practice inhibitors of SphK1 have failed to induce apoptosis whilst the hSphK2 inhibitors, ABC294640 and FTY720, are proving to be more efficacious inhibitors of cell proliferation. Interestingly, the observation that FTY720 binds more efficiently to the unique N-terminal of the SphK2b isoform suggests that the unique SphK2 N-terminal domain may be a more attractive oncotarget such that isoform-specific inhibitors can be designed. Nevertheless, inhibition of SphK2 by some SphK2 inhibitors demonstrated increase in S1P production, and the physiological consequences of this increase are unknown. Adding to the complexity of SphKs role in cancer, aberrant SphK isozyme localization has been cited as potentially contributing to carcinogenesis. Although much insight has been gained from studying SphK isozymes and isoforms in mouse models there are inherent major differences between human and mouse SphK isoforms. More recent attention has been focused on the human SphK isoforms, especially since the discovery that androgen independent prostate cancer cells expressing hSphK1b isoform are refractory to SphK1 inhibitors. Very little is known of the physiology and mechanism of SphK isoforms in culture and clinical samples, such as if one or both isoforms are present in cells and if the physiology of enzymatic regulation is dependent on the dynamic changes in isoform expression and interactions. Understanding the structural conformational plasticity of SphK1 isoforms through homo- and hetero- dimerization and how this relates to normal function and disease will provide more options for SphK oncotarget potential.

In conclusion, although there is great excitement in developing SphK/S1P/S1PR modulators for cancer treatment the intricacies of the SphK pathways are still being unraveled. New knowledge gained from the study of the human SphK isozymes, and in particular human SphK isoforms biology, will provide more understanding of SphKs role in cancer progression, metastasis, and drug resistance, allowing for the development of more specific and effective anti-SphK oncotargets.

## SUPPLEMENTARY FIGURE


