# Supplementary material for: Mammalian sphingosine kinase (SphK) isoenzymes and isoform expression: challenges for SphK as an oncotarget
Source: Oncotarget. 2017 Mar 18;8(22):36898–929. doi: 10.18632/oncotarget.16370 (PMC5482707; doi:10.18632/oncotarget.16370)
Supplement: Supplementary file 1 [file oncotarget-08-36898-s001.pdf]

Mammalian sphingosine kinase (SphK) isoenzymes and isoform expression: challenges for SphK as an oncotarget

Supplementary Material

```
X      N'     MGDTQDSPSGPSEVILCGTFTLVPGTGAPLSLILTRKELLIQTLSLDAQPSPHYSLALADCIGCRSFRGKDPTELGGYLTAAFCPLRGEALGKTIYRQRVSRTFRIEVSRDKEHN-----
D      N'     ------------------------------------------------------------------------------------------------------------------------
BTaur  N'     --------------------------------------M-LGSPIRMGSPRPVYTAKLL----------SDPATAGNDAGAPTAPA-TGGEGEPHSRHR---EARLG-STDKELKSGAAA
HS     N'     ----------------------------------MSAQV-LG-FLRSWTPLPLAA---P----------RGPAAAGNDAGAPAATA-PGGEGEPHSRPC---DARLG-STDKELKAGAAA
Mus    N'     ------------------------------------------------------------------------------------------------------------------------
Rat    N'     --------------------------------------------------MQLDSPPTS----------KDLAASGNDEGAL-TAQ-GGGEGEPHGQSP---DTRLG-NTDKQLHTGAAV

X             ------FNVAKTWAN-RIWEMAEEQGPPSSILPPSRRFLVLLNPFAGTGKASGHFQTHVIPTLTEANATFTLLETERPKQAQELVRDEDLSGWDAIVVMAGDGLLFEVVNGLMERPDWAC
D             --------------------------------------MVLVNPQSGRGQAMAQYNGHVQRMLTEADIPHTLVITERQNHARDLVRSTDLSQWDALIILSGDGLLFEVVNGLMERPDWEK
BTaur         ADSV-RTAPGTPR---QRGEVMDAAGGSRNTLPRPCHVLVLLNPRGGKGKALQLFRSHVQPLLAQADVSFTLMLTERRNHARELVRAEDLRRWDALVVMSGDGLIHEVVNGLMERPDWET
HS            TGSA-PTAPGTPWQREPRVEVMDPAGGPRGVLPRPCRVLVLLNPRGGKGKALQLFRSHVQPLLAEAEISFTLMLTERRNHARELVRSEELGRWDALVVMSGDGLMHEVVNGLMERPDWET
Mus           ---------------------MEPVECPRGLLPRPCRVLVLLNPQGGKGKALQLFQSRVQPFLEEAEITFKLILTERKNHARELVCAEELGHWDALAVMSGDGLMHEVVNGLMERPDWET
Rat           TSSSAGAASVAPCQREPRDLAMQPADCPRGLLPRPCRVLVLLNPRGGKGKALKLFQSRVRPLLEEAEVSFKLMLTERQNHARELVCAEELGHWDALAVMSGDGLMHEVVNGLMERPDWES
                                                    :**:** .* *:*   :. :*   *  :*: ..*: *** ::*::**   :*  ***: :::****:.************

X             AIKKPLAVLPGGSGNALAASVSYYSGHKQASGTKLLNNCTFILCKGQPVPLDLVSLTTSSGRRIFSFLSFAWGLISDVDIESERYRFMGSARFSFGTFVRLTALRTYRGRLSYLPARTSP
D             AIQTPLGILPGGSGNALAASVHHYTRASPVWGEDLLTSCGFLLCKGLVSGLDLISVQLSSGARLFSFLSLAWGFVADVDIESETFRQIGALRFILGTLVRLASLRIYQGKLAYLPAGETD
BTaur         AIQKPLCSLPAGSGNAVAASLNYYAGYEQVTKEDLLTNCTQLLCRRLLAPMDLLSLQTACGQRLFSVLSLAWGFIADVDLESEKFRRLGEMRFTLGACLRLVALRTYRGSLAYLPAETLA
HS            AIQKPLCSLPAGSGNALAASLNHYAGYEQVTNEDLLTNCTLLLCRRLLSPMNLLSLHTASGLRLFSVLSLAWGFIADVDLESEKYRRLGEMRFTLGTFLRLAALRTYRGRLAYLPVGRVG
Mus           AIQKPLCSLPGGSGNALAASVNHYAGYEQVTNEDLLINCTLLLCRRRLSPMNLLSLHTASGLRLYSVLSLSWGFVADVDLESEKYRRLGEIRFTVGTFFRLASLRIYQGQLAYLPVGTVA
Rat           AIQKPLCSLPGGSGNALAASLNYYAGHEQVTNEDLLINCTLLLCCRQLSPMNLLSLHTASGRQLYSVLSLSWGFVADVDLESEKYRSLGEIRFTVGTFFRLASLRIYQGQLAYLPVGKAA
              **:.**  **.*****:***: :*:  . .   .** .*  :**      ::*:*:  :.* :::*.**::**:::***:*** :*  :* ** .*: .**.:** *:* *:***.

X             DSTPESDSHRHNLNSSTDTVNPNVLEDSLLVPLNEPVPPHWTTVTEDQFVLVLLLYQSHLGADLFTAPMVQSPGEGVMQLFYATSRISRASLLKLFLGMEKGSHMGESIPHITNVPVTAF
D             SVSSTGE-----------NL----LNDHLMAPFGQPVPQNWTLVEEREFVLVLAMFQSHLSEDLMAAPGARA-DDGIIHLFYLTAGVSRATLLRLFRAMQSGTHLDCGCAHLVYVRAQAI
BTaur         SRRGPSP-----------ALAPQDPADTHVVPXEQPVPPHWTVVPEQDFVLVLALLHSHLASDLFTAPMGRC-SAGAMHLFYVRAGVSRAMLLRLFLAMEKGRHMECHCPHLVYVPVVAF
HS            SKTPASP-----------VVVQQGPVDAHLVPLEEPVPSHWTVVPDEDFVLVLALLHSHLGSEMFAAPMGRC-AAGVMHLFYVRAGVSRAMLLRLFLAMEKGRHMEYECPYLVYVPVVAF
Mus           SKRPAST------------LVQKGPVDTHLVPLEEPVPSHWTVVPEQDFVLVLVLLHTHLSSELFAAPMGRC-EAGVMHLFYVRAGVSRAALLRLFLAMQKGKHMELDCPYLVHVPVVAF
Rat           SKIPASS------------LAQKGPANTYLVPLEEPVPPHWTVVPEQDFVLVLVLLHTHLSTEMFAAPMGRC-EAGVMHLFYIRAGVSRAMLLRLFLAMQKGKHMDLDCPYLVHVPVVAF
              .    .             :      :  :.*  :*** :** * : :***** : ::**. ::::**  :.   * ::***  : :*** **:**  .*:.* *:     ::. * . *:

X             RLEPLENVGIMTVDGESIPCCSLQGQIHKGLGRIISIPQGP------------------------- C'
D             RLEPLSADGVLTVDGERVHYGPVQAQVHRAAARIIC------------------------------ C'
BTaur         RLEPKDGKGVFTVDGELLISEAVQGQVLPNYFWMVSGYREPPPSLKPQPSPRRKSQQTLPPSERP- C'
HS            RLEPKDGKGVFAVDGELMVSEAVQGQVHPNYFWMVSGCVEPPPSWKPQQMPP---------PEEPL C'
Mus           RLEPRSQRGVFSVDGELMVCEAVQGQVHPNYLWMVCGSRDAPSGRDSRRGPP---------PEEP- C'
Rat           RLEPRNQRGVFSVDGELMVCEAVQGQVHPNYLWMVSGSSDSPSGRDSQRRPP---------PEEPI C'
              **** .  *:::**** :    :*.*:      ::.
```

Supplementary Figure 1: Comparative SphK1 amino acid sequence alignments across multiple species. SphK1 amino acid sequences are evolutionarily conserved across various species from zebrafish to humans. Legend: X = Xenopus tropicalis (frog); D = Danio Rerio (Zebrafish); BTaur = Bos Taurus (cow); HS = Homo sapiens; Mus = Mus musculus; Rat = Rattus norvegicus. N' = N-terminus; C'= C-terminus; * complete amino acid sequence identity across all species compared; : similarity of amino acids across at least 2 species. Sequences aligned using CLUSTAL Omega (1.2.3) multiple sequence alignment tool.
